# Mitochondrial ADP/ATP exchange inhibition: a novel off-target mechanism underlying ibipinabant-induced myotoxicity

**DOI:** 10.1038/srep14533

**Published:** 2015-09-29

**Authors:** Tom J. J. Schirris, Tina Ritschel, G. Herma Renkema, Peter H. G. M. Willems, Jan A. M. Smeitink, Frans G. M. Russel

**Affiliations:** 1 Department of Pharmacology and Toxicology, Radboud University Medical Center, Nijmegen, 6500 HB, The Netherlands; 2 Center for Systems Biology and Bioenergetics, Nijmegen Center for Mitochondrial Disorders, Radboud University Medical Center, Nijmegen, 6500 HB, The Netherlands; 3 Computational Discovery and Design Group, Center for Molecular and Biomolecular Informatics (CMBI), Radboud University Medical Center, Nijmegen, 6500 HB, The Netherlands; 4 Department of Pediatrics, Radboud University Medical Center, Nijmegen, 6500 HB, The Netherlands; 5 Department of Biochemistry, Radboud University Medical Center, Nijmegen, 6500 HB, The Netherlands

## Abstract

Cannabinoid receptor 1 (CB1R) antagonists appear to be promising drugs for the treatment of obesity, however, serious side effects have hampered their clinical application. Rimonabant, the first in class CB1R antagonist, was withdrawn from the market because of psychiatric side effects. This has led to the search for more peripherally restricted CB1R antagonists, one of which is ibipinabant. However, this 3,4-diarylpyrazoline derivative showed muscle toxicity in a pre-clinical dog study with mitochondrial dysfunction. Here, we studied the molecular mechanism by which ibipinabant induces mitochondrial toxicity. We observed a strong cytotoxic potency of ibipinabant in C2C12 myoblasts. Functional characterization of mitochondria revealed increased cellular reactive oxygen species generation and a decreased ATP production capacity, without effects on the catalytic activities of mitochondrial enzyme complexes I–V or the complex specific-driven oxygen consumption. Using *in silico* off-target prediction modelling, combined with *in vitro* validation in isolated mitochondria and mitoplasts, we identified adenine nucleotide translocase (ANT)-dependent mitochondrial ADP/ATP exchange as a novel molecular mechanism underlying ibipinabant-induced toxicity. Minor structural modification of ibipinabant could abolish ANT inhibition leading to a decreased cytotoxic potency, as observed with the ibipinabant derivative CB23. Our results will be instrumental in the development of new types of safer CB1R antagonists.

Nowadays, overweight and obesity are worldwide one of the greatest health challenges^1^. Compared to other modifiable cardiovascular risk factors, obesity is still a poorly understood condition for which treatment options remain elusive^2^. Overstimulation of the endocannabinoid system, which plays an important role in metabolism and energy balance, has been associated with obesity^3^,^4^. Signalling in this system is mainly mediated through both centrally and peripherally expressed cannabinoid-1 receptors (CB1R)^5^,^6^. CB1R antagonists appeared to be beneficial in rodent models of obesity, leading to reduced food intake and body weight^7^,^8^. Similar effects were also observed in clinical trials with rimonabant, the only approved CB1R antagonist for therapeutic use^9^. The drug was, however, rapidly withdrawn from the market after the observation of serious neuropsychiatric side effects, which could mainly be attributed to central nervous system effects by rimonabant’s ability to pass the blood-brain barrier^10^.

The demand for a therapy to counteract obesity, combined with multiple other beneficial effects on plasma triglyceride levels, fasting insulin and glucose levels, and β-cell function in diabetes, has led to the search for peripherally restricted CB1R antagonists^4^,^7^. This was based on the observation that reduction of food intake could also be accomplished through a mechanism independent of central CB1R occupancy, thereby avoiding the neuropsychiatric side effects^7^,^8^,^11^. These effects may be partially explained by the capacity of peripheral CB1R antagonists to lower leptin expression and secretion by adipocytes, combined with an increased renal leptin clearance^12^. Consequently, hyperleptinemia observed with obesity is reversed, which leads to reduced hypothalamic endocannabinoid levels, thereby indirectly affecting central appetite regulation^13^.

Compared to rimonabant, which is a 1,5-diarylpyrazole derivative, the 3,4-diarylpyrazoline ibipinabant (S-SLV-319) showed substantially lower levels of centrally occupied CB1R (11% vs. 80%), which might be due to a lower passage of the blood-brain barrier^11^,^14^. Therefore, ibipinabant was used as a template for the development of several novel 3,4-diarylpyrazoline CB1R antagonists^8^,^11^.

During preclinical development of ibipinabant, however, striated-muscle toxicity was observed in a dog-study, which was shown to be CB1R independent^15^. The authors attributed the evident mitochondrial dysfunction to the inhibition of flavin-containing enzymes, as concluded from a metabolic pattern matching ethylmalonic-adipic aciduria in humans^15^. However, the exact mechanism underlying ibipinabant-induced myopathy remains unresolved.

Here, we unravelled the effect of ibipinabant on mitochondrial function in C2C12 myoblasts. We found increased generation of cellular reactive oxygen species (ROS) and decreased ATP production capacity, which was linked to an increased mitochondrial membrane potential. By *in silico* off-target modelling we could predict both the voltage-dependent anion channel (VDAC) and the adenine nucleotide translocase 1 (ANT1) as the potential molecular site of ibipinabant inhibition. This *in silico* prediction was experimentally verified by a decreased mitochondrial ATP/ADP exchange. Moreover, these effects could be abolished by minor structural modification of ibipinabant.

## Results

### Ibipinabant is a potent inducer of cytotoxicity in C2C12 myoblasts accompanied by mitochondrial dysfunction

**Figure 1:**
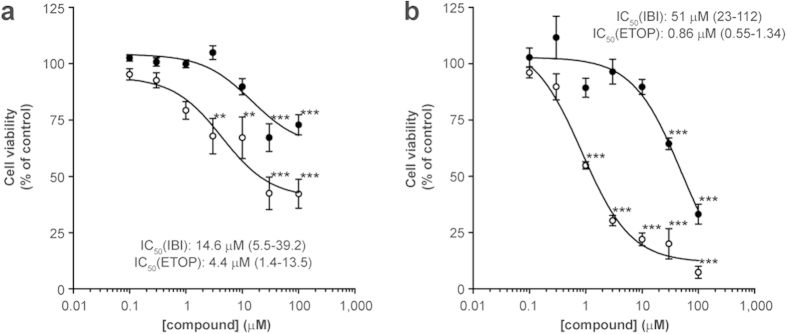
Ibipinabant-induced cytotoxicity in C2C12 myoblasts after 24 and 48 hours exposure. The number of cells was determined after 24 **(A)** and 48 **(B)** hours exposure of C2C12 myoblasts to increasing ibipinabant (•) concentrations. As a positive control, cells were exposed to etoposide (◦), a known inducer of mitochondria-mediated cell death. Cell viability was determined as the percentage of cells compared to vehicle control. Statistical analysis: one-way ANOVA with Dunnett’s post hoc analysis was applied to compare values to vehicle control **p < 0.01, ***p < 0.001. Mean ± SEM; n = 18 (3 independent experiments); IC_50_ values are plotted as mean with 95%-CI.

To gain more insight into the mechanisms underlying ibipinabant-induced myotoxicity, we used C2C12 murine myoblasts as a cell model. Already after 24 hours of exposure to increasing concentrations of ibipinabant, cell viability was significantly (P=1.61·10^-7^) decreased to 73 ± 5% at the highest concentration tested (100 μM, Fig. 1A). After 48 hours of exposure only 33 ± 4% of the cells remained viable at this concentration (Fig. 1B). The validity of our model was confirmed by the potent inhibition of cell viability by the known mitochondrial toxicant etoposide. At the highest concentration of 100 μM 42 ± 6% cells remained viable after 24 hours (Fig. 1A), which further decreased to 7 ± 3% after 48 hours (Fig. 1B).

**Figure 2:**
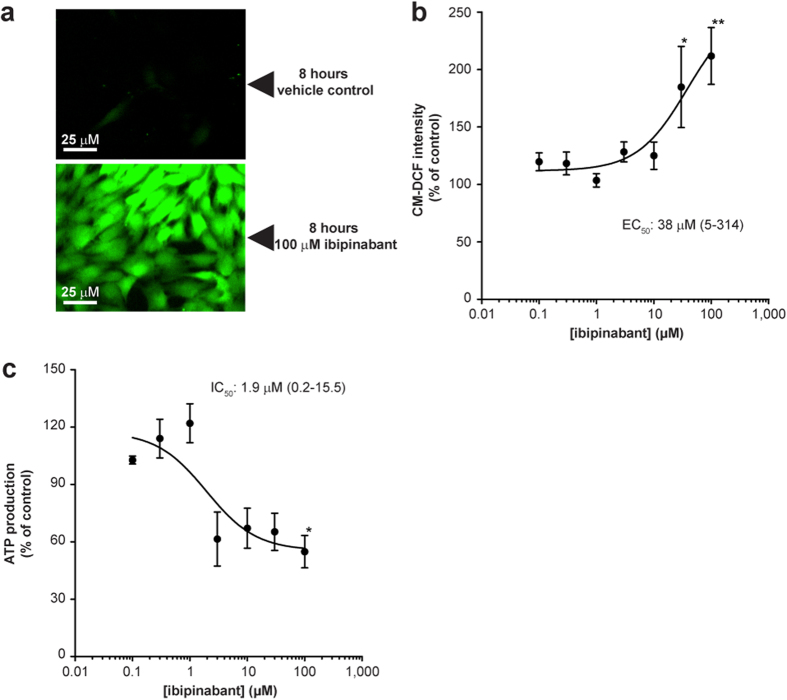
Ibipinabant leads to increased generation of reactive oxygen species and decreased mitochondrial ATP production. **(A)** ROS generation was measured after 8 hours exposure of C2C12 myoblasts to ibipinabant using CM-H_2_DCFDA. **(B)** Followed by quantification of the CM-DCF intensity compared to vehicle control (640 ± 230 IU/cellular pixel) for increasing ibipinabant concentrations. Mean ± SEM; n = 12 (3 independent experiments); IC_50_ values are plotted as mean with 95%-CI. **(C)** Maximal ATP production was measured in permeabilised C2C12 cells after 4 hours of incubation with various ibipinabant concentrations. Data are expressed as percentage of vehicle (22 ± 6 nmol/h/mU CS). Mean ± SEM; n = 3 independent experiments; IC_50_ values are plotted as mean with 95%-CI. Statistical analysis: one-way ANOVA with Dunnett’s post hoc analysis was applied to compare values to vehicle control *p < 0.05, **p < 0.01.

Next, we determined the effects of ibipinabant on mitochondrial function by measuring cellular ROS generation. A more than 2-fold increase could already be observed after 8 hours exposure with the highest ibipinabant concentration of 100 μM compared to the vehicle treated C2C12 myoblasts (Fig. 2A,B). We also investigated maximal mitochondrial ATP production capacity after permeabilisation of the C2C12 myoblasts (Fig. 2C). As indicated by the low IC_50_ value, a rapid decrease of ATP production capacity was observed after 4 hours incubation with increasing concentrations of ibipinabant (Fig. 2C).

### Ibipinabant exposure affects mitochondrial coupling without alteration of complex I-V activity or respiratory capacity

A possible explanation for a decrease in ATP production capacity, accompanied by an increased generation of cellular ROS, could be inhibition of one of the enzyme complexes of the respiratory chain (complexes I–V). To examine this assumption we measured the oxygen consumption in C2C12 myoblasts after 4 hours of pre-incubation with ibipinabant (1–100 μM). In intact cells we did not observe a statistically significant effect of ibipinabant (see Supplementary Fig. S1 online). To investigate whether one of the specific complexes might be affected by ibipinabant under maximal stimulation, we used complex specific substrates to measure oxygen consumption in permeabilised C2C12 myoblasts (see Supplementary Fig. S1 online). However, none of the specific complex-driven respiratory rates was different from control. To be able to exclude any influence of 4 hours ibipinabant treatment on these complexes we also determined their catalytic activity and in agreement with the absence of effects on oxygen consumption, no effects could be observed on the enzyme activities (see Supplementary Fig. S1 online).

**Figure 3:**
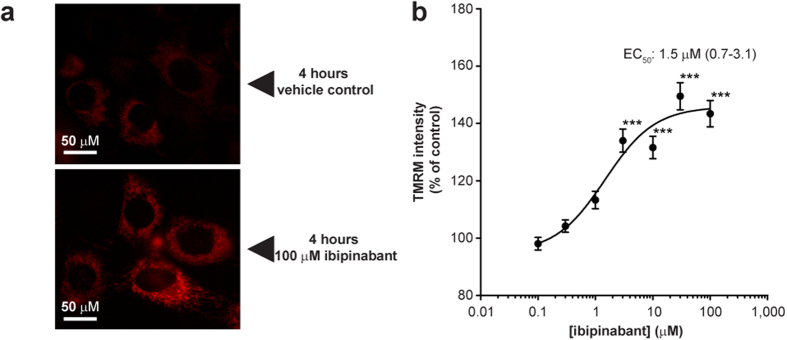
Mitochondrial membrane potential is increased after 4 hours ibipinabant exposure. (**A**) Mitochondrial membrane potential was measured by loading mitochondria with the cationic dye tetramethylrodamine methyl ester (TMRM) after 4 hours exposure of C2C12 myoblasts to ibipinabant. (**B**) Followed by quantification of the TMRM intensity compared to vehicle control (13.1 ± 0.3 IU/cellular pixel) at increasing ibipinabant concentrations. Statistical analysis: one-way ANOVA with Dunnett’s post hoc analysis was applied to compare values to vehicle control ***p  <0.001. Mean ± SEM; n = 60–80 (4 independent experiments); IC_50_ values are plotted as mean with 95%-CI.

Uncoupling of oxidative phosphorylation is one of the explanations for the absence of an effect of ibipinabant on the enzyme complexes of the respiratory chain with a decreased ATP production^16^. The respiratory chain and mitochondrial ATP production, which together are referred to as oxidative phosphorylation, are coupled through the mitochondrial membrane potential (Ψ_m_ ), providing the proton motive force needed for ATP production at mitochondrial complex V. Indeed, Ψ_m_ appeared to be increased after 4 hours of incubation with 100 μM ibipinabant (Fig. 3A). An increase in Ψ_m_ could already be observed at low ibipinabant concentrations (EC_50_ 1.5 95%−CI: 0.7–3.1 μM) (Fig. 3B). Such an increased Ψ_m_ could be indicative of a decreased function of complex V in intact mitochondria, following from the decreased ATP production capacity. Together with the absence of an effect on the catalytic activity of complex V, under excess substrate conditions (see Supplementary Fig. S1 online), these findings point at a decreased ADP availability in the mitochondrial matrix.

### Ibipinabant inhibits mitochondrial ANT-mediated ADP/ATP exchange

To address this possibility, *in silico* off-target predictions were performed to search for similarities between protein-ligand binding sites with the structure-based pharmacophore method KRIPO, possibly identifying off-targets involved in mitochondrial ADP/ATP exchange^17^. Each protein-ligand binding site is defined via the ligand of the complex followed by translation of the properties of the surrounding amino acids of the proteins into pharmacophore features, which are stored in bit strings called fingerprints. The fingerprints allow fast computational comparison of all publically available structures in the Protein Data Bank (pdb)^18^. Since for CB1R no experimental structure is available, a homology model of the receptor was build. From the list of similar targets obtained by KRIPO, targets that could play a key role mitochondrial ADP/ATP exchange (adenine nucleotide translocase 1 (ANT1), voltage-dependent anion channel (VDAC), and mitochondrial complex V) were manually selected.

**Figure 4:**
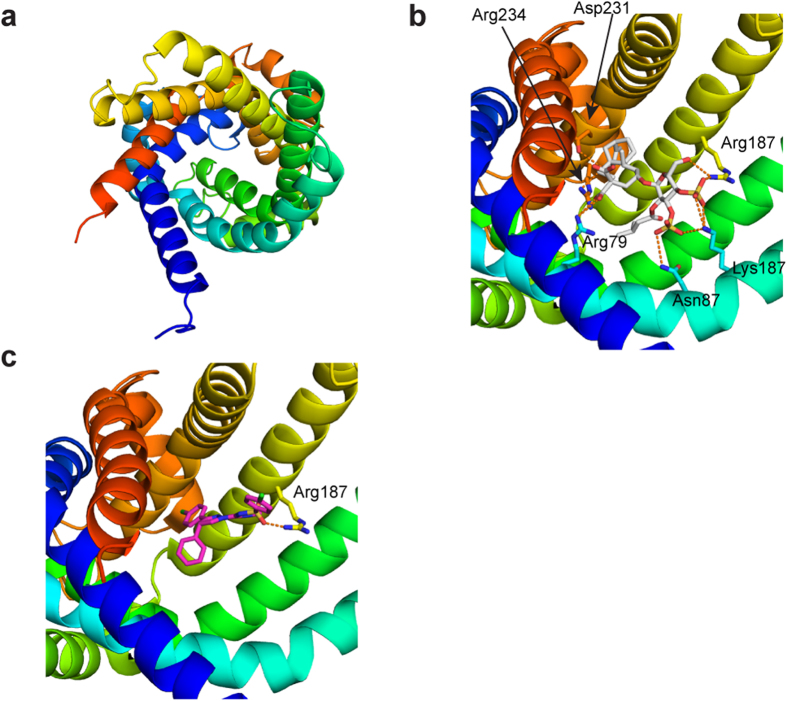
X-ray structure of ANT1 compared to a possible ibipinabant docking pose. (**A**) X-ray structure of ANT1 (pdb-code 2c3e) displayed as ribbon are colored from blue (N-terminus) to red (C-terminus) (**B**) ANT1 with a known inhibitor carboxyatractyloside (grey). (**C**) A possible docking pose of ibipinabant (pink) to ANT1.

Of these three targets, an effect of ibipinabant on mitochondrial complex V could be excluded from our previous observations (see Supplementary Fig. S1 online). To investigate whether ibipinabant can actually fit into ANT1, the drug was docked into the binding pocket of the protein binding sites (Fig. 4). In the co-crystallised complex the binding site is occupied by the inhibitor carboxyatractyloside (Fig. 4B), a ten times more potent inhibitor compared to atractyloside, which was used in the experiments described below^19^. These docking runs suggested that binding of ibipinabant to ANT1 appears likely, and a possible binding mode is illustrated (Fig. 4C). Whether ibipinabant would fit into VDAC could not be determined, because reliable docking was not possible due to the structural properties of the channel, *e.g.* having a large pore allowing unselective transport of many compounds.

**Figure 5:**
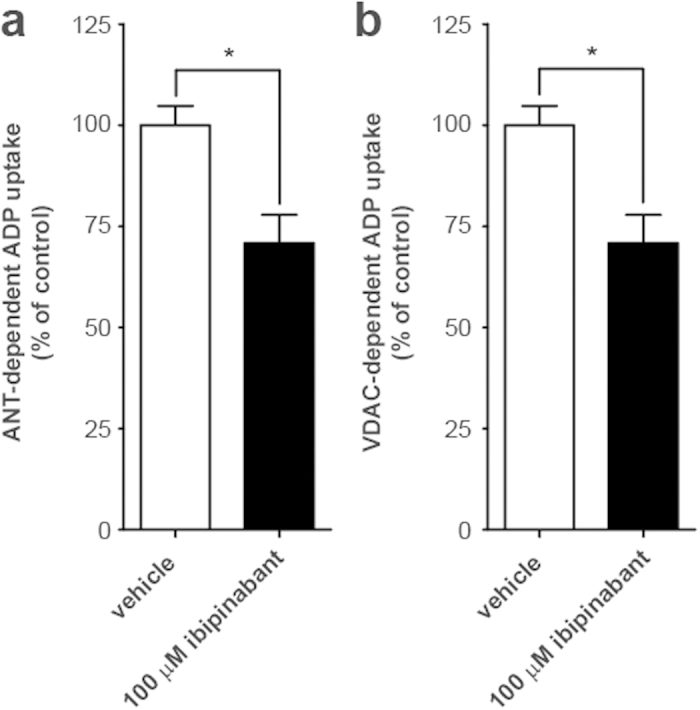
Ibipinabant-induced inhibition of ANT- and VDAC-dependent mitochondrial ADP uptake. Mitochondrial uptake of radiolabeled ADP was measured in bovine heart mitochondria with and without the model inhibitors **(A)** atractyloside (50μM) and **(B)** DIDS (500 μM) to determine ANT- and VDAC-dependent ADP uptake, respectively. The effects of direct administration of 100 μM ibipinabant were investigated under both conditions. Values presented are expressed as the percentage of vehicle control (delta cumulative counts, with and without model inhibitor): 1.61·10^4 ^± 0.10·10^4^ for ANT, and 1.70·10^4 ^± 0.10·10^4^ for VDAC. Statistical analysis: Student’s t-test was applied to compare values to vehicle control *p < 0.05. Mean ± SEM; n = 4 independent experiments.

The *in silico* predictions were experimentally verified by measuring ADP uptake into isolated bovine heart mitochondria (Fig. 5). This resulted in a significant (p = 0.041) decrease of mitochondrial VDAC-dependent ADP import after direct ibipinabant application (Fig. 5B). A similar effect could be observed on the ANT-dependent mitochondrial ADP uptake (Fig. 5A). Such an effect on both transmembrane transporters could be explained by the formation of a complex spanning the inner and outer mitochondrial membrane, as described previously^20^. The observations in intact bovine heart mitochondria (containing both ANT and VDAC), could therefore not distinguish which of these transporters contributed to the effects of ibipinabant on ATP production and Ψ_m_.

**Figure 6:**
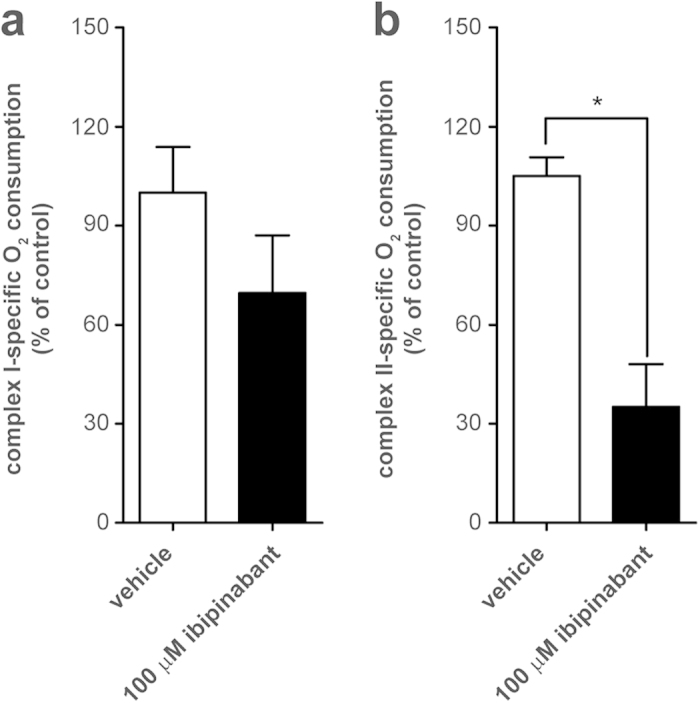
Ibipinabant-induced inhibition of respiratory capacity in mitoplasts. C2C12 mitoplasts were obtained by differential centrifugation of (sub)cellular fractions. Maximal respiratory capacity was determined after acute exposure to 100 μM ibipinabant either by stimulation of **(A)** CI- or **(B)** CII-driven respiration. Effects of VDAC were excluded by the addition of 250 μM DIDS, resulting in complete inhibition of VDAC-dependent respiration. The data presented are expressed as percentage of vehicle-treated control (pmolO_2_/s/mg protein): 14 ± 2 for CI, 25 ± 1 for CII. Statistical analysis: Student’s t-test was applied to compare values to vehicle control *p < 0.05. Mean ± SEM; n = 3 independent experiments.

To investigate whether the observed ibipinabant-induced decrease in mitochondrial ADP/ATP exchange was due to inhibition of either ANT or VDAC, C2C12 mitoplasts (mitochondria without outer membrane) were isolated to exclude any effects of VDAC, followed by determination of the maximal CI- and CII-specific respiratory rates (Fig. 6). Although CI-specific respiration was only moderately affected after direct ibipinabant addition, a significant (P=0.038) inhibition was found for CII-specific respiratory rates. These effects were also in line with the tendency towards a decreased routine respiration (see Supplementary Fig. S1 online), which also indicated that mitoplasts were more sensitive than intact mitochondria to ibipinabant-induced respiratory inhibition. It also provided a firm indication that ibipinabant mainly reduced respiration by inhibition of the ANT-mediated ADP/ATP exchange.

### Minor structural modification of ibipinabant abolishes ANT and VDAC inhibition

**Figure 7:**
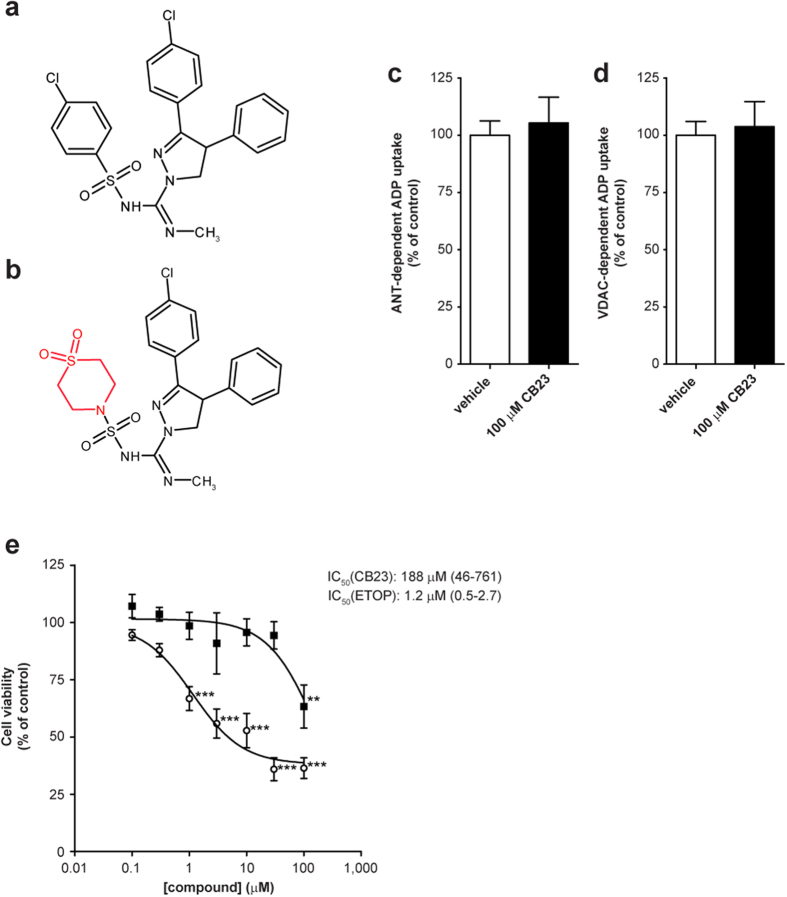
Ibipinabant derivative 23 (CB23) does not inhibit ANT- and VDAC-dependent mitochondrial ADP uptake and has a low cytotoxic potency. The effect of structural modification of (**A**) ibipinabant on mitochondrial ADP uptake was investigated using (**B**) CB23. This derivative was obtained by replacing the chlorophenyl group by a 1,1-dioxo-thiomorpholine group (marked in red). The effects of direct administration of 100 μM CB23 were investigated both for (**C**) ANT- and (**D**) VDAC-dependent mitochondrial ADP uptake. Experimental procedures and calculations were as described in Fig. 6. Statistical analysis: Student’s t-test was applied to compare values to vehicle control, no significant differences were observed. Mean ± SEM; n = 5 independent experiments. (**E**) The number of cells was determined after 24 hours exposure of C2C12 myoblasts to increasing CB23 (■) concentrations. As a positive control, cells were exposed to etoposide (◦), a known inducer of mitochondria-mediated cell death. Cell viability was determined as described in Fig. 1. Statistical analysis: one-way ANOVA with Dunnett’s post hoc analysis was applied to compare values to vehicle control **p < 0.01, ***p < 0.001. Mean ± SEM; n = 18 (3 independent experiments); IC_50_ values are plotted as mean with 95%-CI.

Finally, we compared the inhibitory effects of ibipinabant on mitochondrial ADP import with the closely related 3,4-diarylpyrazoline derivative, CB23 (Fig. 7B). Replacement of the chlorophenyl group of ibipinabant with a 1,1-dioxo-thiomorpholine group (Fig. 7A,B), completely abolished the inhibitory effect on VDAC- and ANT-dependent mitochondrial ADP import (Fig. 7C,D). Moreover, CB23 exhibited a significantly lower cytotoxic potential as compared to ibipinabant (IC_50_ values: 188 vs 17 μM, P=0.034, for 95%-CI see Fig. 7E). These findings also support that the cytotoxic effects observed with ibipinabant are related to inhibition of the ANT-dependent ADP/ATP exchange.

## Discussion

We report that ibipinabant inhibits the mitochondrial ADP/ATP exchange ratio and thereby provide a plausible mechanism for the previously observed striated muscle toxicity in a pre-clinical dog study^15^. We could show that these effects occur after direct exposure, leading to a rapid decrease in ATP production, which we hypothesize to be due to decreased ADP content in the mitochondrial matrix. The accompanied reduction in use of protons from the mitochondrial inner membrane space by complex V leads to an increase in Ψ_m_, if the complexes of the oxidative phosphorylation chain retain their outward proton motive force. Both effects could indeed be observed after treating C2C12 cells for 4 hours with ibipinabant. An increased Ψ_m_ combined with a decreased ATP production are both characteristic for the initial phase of apoptosis^21^. This phase is followed by a collapse of the mitochondrial membrane potential and decreased oxygen consumption, explaining the increased cellular ROS levels after 8 hours of ibipinabant exposure, eventually initiating the cytotoxicity observed after 24 and 48 hours. Interestingly, the increased ROS levels and observed cytotoxicity resulted in much higher IC_50_ values, as compared to the effects on ATP production capacity and mitochondrial membrane potential. This difference could be explained by the presence of compensatory mechanisms, such as up-regulation of cellular anti-oxidant systems affecting the former observations, which further demonstrates the major role of altered ADP/ATP exchange, and not the increased ROS levels, in the cytotoxic effects of ibipinabant.

Inhibition of mitochondrial ADP/ATP exchange can also explain the decreased fatty acid oxidation with concomitant lipid accumulation in the muscles, which was associated with ibipinabant-induced myotoxicity in the dog^15^. Decreased mitochondrial oxidative metabolism due to limited supply of ADP, will likely also decrease fatty acid oxidation. This could lead to accumulation of fatty acids, particularly in tissues like muscle, which heavily rely on oxidative metabolism and particularly fatty acid oxidation^22^. Although our results are in agreement with the effects described in dogs, it should be noted that C2C12 myoblasts express a functional CB1R, whereas the dog striated muscle does not^15^,^23^. However, previous studies have shown that stimulation, and not inhibition, of CB1R induced mitochondrial dysfunction. The use of AM251, a full CB1R antagonist like ibipinabant, completely reversed the CB1R-mediated mitochondrial dysfunction under these conditions. This points to a protective rather than a toxic effect of CB1R blockade, excluding a role of CB1R with regard to our observations^24^,^25^,^26^.

Although high ibipinabant concentrations were used in this study, they are in the range of the micromolar peak plasma concentrations previously observed in mice after an oral dose of 100 mg/kg for three consecutive days^27^. Furthermore, the observed muscle toxicity in dogs occurred after chronic exposure of a 3-fold higher daily dose (300 mg/kg for 13 weeks), which due to the lipophilic nature of ibipinabant may even have led to accumulation in the muscles^15^.

Based on the *in silico* off-target predictions and docking runs, ANT1 inhibition by ibipinabant is the most likely explanation for the decreased ADP import into the mitochondria, although an inhibitory effect on the VDAC channel cannot be fully excluded. They both play a pivotal role in mitochondrial ADP/ATP exchange^28^, and are hypothesized to interact with each other to form a complex, in which ANT embedded in the mitochondrial inner membrane is probably directly connected with VDAC located in the outer membrane^20^. However, using mitoplasts, we could separate the effects on both proteins and demonstrate that ibipinabant indeed inhibited ANT-dependent respiratory capacity. This is confirmed by a mouse ANT-/- knockout model, which also demonstrates mitochondrial dysfunction and mitochondrial myopathies^29^,^30^. Although ANT seems to be a more probable binding site of ibipinabant than VDAC, mutation studies or co-crystallisation should provide the final proof.

The absence of mitochondrial ADP import inhibition by the ibipinabant derivative CB23 emphasizes that minor structural modifications could determine the occurrence of off-target muscle toxicity. Previously, we selected CB23 from a series of 3,4-diarylpyrazoline CB1R antagonists to proof the concept that its properties as a substrate of the efflux transporters P-glycoprotein and BCRP (breast cancer resistance protein), limits brain penetration and can be exploited to develop peripherally acting antagonists^31^,^32^. The observed effects of ibipinabant on mitochondrial ADP/ATP exchange could also be relevant for the development of other new CB1R antagonists based on ibipinabant^8^,^11^. Other new, peripherally restricted, CB1R antagonists are derivatives of ibipinabant in which either the 4,5-dihydropyrazole moiety or the central N-methyl group was replaced with polar pendants^8^,^11^. Of the latter category, compounds JD5006 and JD5037, seemed to have the best efficacy in inhibition of CB1R and food intake^8^,^11^,^27^. Both antagonists showed a strong inhibitory potency against the CB1R, which was highest for JD5037 that recently received the status of investigational new drug. It might therefore be needed to carefully consider their effects on mitochondrial ADP/ATP exchange, when these compounds are further evaluated for therapeutic use. Moreover, based on the similarities between the CB1R and ANT1 binding sites, other CB1R antagonists could also have the potential to inhibit ANT1.

In conclusion, we revealed the mechanism underlying the off-target effect of ibipinabant-induced myotoxicity. This mechanism may be relevant for newly developed peripherally restricted CB1R antagonist, and an altered mitochondrial ADP/ATP exchange should be taken into consideration during further development of these compounds.

## Methods

### Compounds

Ibipinabant (S-SLV 319) was ordered at Cayman Chemical (Ann Arbor, MI). A racemic mixture of 3-(4-chlorophenyl)-4-phenyl-4,5-dihydro-1*H*-pyrazole-1-carboxamide derivate 23 (CB23) was kindly provided by Abbott Products GmbH (Hannover, Germany). Etoposide, 4,4′-diisothiocyanatostilbene-2,2′-disulfonicacid disodium salt hydrate (DIDS), and atractyloside sodium salt were from Sigma Aldrich (Zwijndrecht, The Netherlands).

### Cell culture

C2C12 murine myoblasts (CRTL 1772) were obtained from American Type Culture Collection (Wesel, Germany). C2C12 cells were maintained in Dulbecco’s modified Eagle’s medium with Glutamax I formulation containing 25 mM glucose and 25 mM HEPES supplemented with 10% (v/v) fetal calf serum (MP Biomedicals, Santa Ana, CA) at 37 °C in a humidified atmosphere of 5% CO_2_.

### Analysis of cell death

C2C12 myoblasts were seeded in 384-wells black/clear imaging plates at a density of 4,000 cells/well, 24 hours prior to ibipinabant treatment. A 1,000× concentrated serial √10-dilution in DMSO was made for ibipinabant and the positive control etoposide. Immediately before use, ibipinabant and etoposide stock solutions were diluted 100× in phosphate buffered saline (PBS) and 10× in the culture medium, resulting in a final DMSO concentration of 0.1% (v/v). Four replicates of each compound were tested up to maximum solubility (100 μM). After 24 h and 48 h exposure, nuclei were stained for cell viability analysis using Hoechst 33342. After 20 minutes of staining at 37 °C, fluorescence was imaged on a BD Pathway 855 high-throughput microscope (Becton Dickinson (BD) Bioscience, Breda, The Netherlands). Followed by the analysis of the number of nuclei using Cellprofiler^33^.

### Analysis of cellular reactive oxygen species

C2C12 myoblasts were seeded in 384-wells black/clear imaging plates as described for analysis of cell death. After 8 hours of ibipinabant exposure cells were loaded with 10 μM CM-H_2_DCFDA in HEPES-Tris (HT) buffer (132 mM NaCl, 10 mM HEPES, 4.2 mM KCl, 1 mM MgCl_2_, 1 mM CaCl_2_ and 25 mM D-glucose, adjusted to pH 7.4 with Tris) for 20 minutes at 37 °C. Next, the cells were washed twice with HT buffer and imaged on a BD Pathway 855 high-throughput microscope. CM-DCF images were processed using Image Pro Plus 6.3 software (Media Cybernetics, Silver Spring, MD). A mask of the images was made to separate the cellular pixels from the background; afterwards average CM-DCF intensity per cellular pixel was determined.

### Mitochondrial ATP production capacity

After 4 hours ibipinabant exposure, C2C12 cells were resuspended in PBS with 3% bovine serum albumin, to a final density of 10·10^6^ cells/mL. Next, mitochondrial ATP production from pyruvate was measured with 1.10^5^ cells per incubation in the presence of 20 μg digitonin/10^5^ cells in duplicate as described previously^34^,^35^,^36^. All values were normalized to the activity of the mitochondrial matrix enzyme, citrate synthase, to correct for differences in mitochondrial mass. Citrate synthase was determined spectrophotometrically in duplicate as described before^35^.

### Analysis of cellular and mitochondrial respiration

For measurement of the cellular respiration rate, 1.5·10^6^ C2C12 cells were resuspended in mitochondrial respiration medium MiR05 after 4 hours ibipinabant exposure, and transferred to the thermostated (37 °C) chamber of an Oxygraph-2k equipped with Datlab 5 recording and analysis software (Oroboros Instruments, Innsbruck, Austria). Mitochondrial respiration driven by the separate respiratory chain complexes was measured after digitonin permeabilisation (10 μg/1·10^6^ cells) of the cell membrane in MiR05 containing complex-specific substrates and 4 mM ADP^37^. Glutamate (10 mM) plus malate (2 mM) were used as substrates for complex I (CI), succinate (10 mM) for CII, and ascorbate (2 mM) plus TMPD (0.5 mM) for CIV. CIII-driven respiration was measured in the presence of glycerophosphate (20 mM) and flavine adenine dinucleotide (10 μM). Rotenone (0.5 μM) and atpenin A5 (50 nM) were added to inhibit CI and CII, respectively. Cytochrome c (10 μM) was added to check the integrity of the mitochondrial outer membrane. Finally, antimycin A (2,5 μM) was added to completely block CIII-driven respiration. Mitoplasts were isolated from C2C12 myoblasts to determine CI- and CII-specific respiration. Briefly, pellets of 10·10^6^ cells were resuspended in 10 mM Tris-HCl and pottered, and sucrose was added (215 mM). The lysate was cleared of unbroken cells by centrifugation (10 minutes 600 *g*), after which the supernatant containing the mitochondria was pelleted at 14,000 *g* for 10 minutes. Next, mitochondria were resuspended in hypotonic buffer (2% essential fatty acid free BSA, 20 mM K_2_HPO_4_, pH 7.4) and allowed to swell for 30 minutes, as described previously^38^, and subsequently pottered to remove the outer membrane. Mitoplasts were obtained from the lysate by centrifugation at 14,000 *g* for 15 minutes, followed by resuspension of the pellet in MiR05. At the start of the experiment DIDS (250 μM) was added, just after the addition of cytochrome c, to exclude any influence of VDAC, and followed by determination of the CI- and CII-specific respiration as described above. Respiratory rates were normalized to mg protein in the mitoplast fractions as determined in duplicate by a Biorad protein assay (Biorad), after three sequential freeze thaw cycles to disrupt the inner membrane. Finally, all respiratory rates were corrected for non-mitochondrial respiration by subtraction of the residual respiratory rates after rotenone or antimycin A addition.

### Catalytic capacity of individual OXPHOS complexes

After 4 hours ibipinabant exposure, C2C12 pellets of 20·10^6^ cells were snap frozen in liquid nitrogen and kept at −80 °C until use. For mitochondrial preparations, cells were resuspended in 10 mM Tris-HCl and pottered, and sucrose was added (215 mM). The lysate was cleared of unbroken cells by centrifugation (10 minutes 600 *g*) after which the supernatant containing the mitochondria was pelleted at 14,000 *g* for 10 minutes, resuspended in 10mM Tris-HCl (pH 7.6), and snap frozen in aliquots. Catalytic capacity of OXPHOS complexes was measured spectrophotometrically in duplicate, as described previously^39^,^40^,^41^. Complex I-IV values were normalized to the activity of the mitochondrial matrix enzyme, citrate synthase, to correct for differences in mitochondrial mass. Citrate synthase was determined spectrophotometrically in duplicate as described before^35^. Complex V values were normalized to cytochrome c oxidase (COX, complex IV) activity.

### Analysis of mitochondrial membrane potential

C2C12 cells (5.0·10^4^) were seeded in 35 mm Fluorodishes (World Precision Instruments, Sarasota, FL), 24 hours prior to ibipinabant exposure. To measure mitochondrial membrane potential after 8 hours ibipinabant treatment, cells were loaded with 100 nM tetramethylrhodamine methyl ester (TMRM) for 25 minutes at 37 °C as described previously^42^. Next, cells were maintained in HT buffer, and images were obtained using a temperature-controlled chamber attached to a stage of an inverted microscope (Axiovert 200M, Carl Zeiss, Jena, Germany) equipped with a ×63, 1.25 NA Plan NeoFluar oil immersion objective. TMRM intensity analysis was performed on background corrected images, which were masked with a binarised image for mitochondrial morphology using Image Pro Plus 6.3 software.

### *In silico* off-target prediction

Since no experimental structure of CB1R is available, a homology model was built with the automated protocol of Yasara (version 13.9.8, www.yasara.org)^43^ and pdb-code 3v2y as template^44^. The co-crystallised ligand ML5 of the pdb-entry 3v2y complex is used to define the binding site of CB1R for KRIPO and calculate a structure based fingerprint bit string^17^. This bit string was compared to the PDB (version March 2014)^45^.

### Docking of ibipinabant

The structure of bovine mitochondrial ADP/ATP carrier isoform 1 (pdb-code 2c3e)^46^ was used for docking of ibipinabant using Molecular Operating Environment (MOE) (version 2013.0802, Chemical Computing Group Inc, Montreal, Canada). The structure preparation and protonate 3D protocol of MOE was applied before the docking. Docking was performed using standard settings. Ibipinabant was prepared in MOE by assigning PEOE charges and energy minimization.

### Mitochondrial ADP uptake assay

ADP transport across the mitochondrial membrane was measured in isolated bovine heart mitochondria, as described before^47^,^48^. Briefly, 10 uL (equal to 130 μg protein) mitochondrial pellet was resuspended in ADP import buffer (250 mM sucrose, 20 mM HEPES, pH 7.2, 10 mM KCl, 5 mM succinate, 3 mM KH_2_PO_4_, 1.5 mM MgCl_2_, 1 mM EGTA, and 5 μM rotenone) with or without the adenine nucleotide translocase inhibitor atractyloside (50 μM) to inhibit ANT-dependent ADP uptake, or with or without DIDS (500 μM) to inhibit VDAC-dependent ADP uptake. [14C]ADP (1 μCi) was added to the mitochondrial suspension and incubated for 10 min on ice. After washing two times with ADP import buffer, the samples were resuspended in scintillant and quantified using a scintillation counter (PerkinElmer Tri-Carb^®^ 2900TR, Perkin Elmer, Waltham, MA). ANT- and VDAC-dependent ADP transport activity was determined by the difference in counts between samples that respectively were or were not pre-incubated with atractyloside or DIDS. ADP import was decreased to 13.8 ± 1.1% and to 8.4 ± 0.8% respectively with atractyloside or DIDS alone.

### Statistical analysis

Curve-fitting and statistical analysis was performed using GraphPad prism 5.02 software (GraphPad Software Inc., San Diego, CA). Unless indicated otherwise, all results are presented as mean ± SEM. Differences between groups were tested using one-way ANOVA analysis with appropriate post-hoc tests, unless indicated otherwise.

## Additional Information

**How to cite this article**: Schirris, T. J. J. *et al.* Mitochondrial ADP/ATP exchange inhibition: a novel off-target mechanism underlying ibipinabant-induced myotoxicity. *Sci. Rep.* **5**, 14533; doi: 10.1038/srep14533 (2015).

## Supplementary Material

Supplementary Information
