# Supplementary material for: Mitochondrial ADP/ATP exchange inhibition: a novel off-target mechanism underlying ibipinabant-induced myotoxicity
Source: Sci Rep. 2015 Sep 29;5:14533. doi: 10.1038/srep14533 (PMC4586513; doi:10.1038/srep14533)
Supplement: Supplementary Information [file srep14533-s1.pdf]

Supplementary Information

Mitochondrial ADP/ATP exchange inhibition: a novel off-target mechanism underlying ibipinabant-induced myotoxicity

Tom J.J. Schirris[1,2], Tina Ritschel[3], G. Herma Renkema[2,4], Peter H.G.M. Willems[2,5], Jan A.M. Smeitink[2,4], Frans G.M. Russel[1,2,*]

[1] Department of Pharmacology and Toxicology, Radboud University Medical Center, Nijmegen, 6500HB, The Netherlands

[2] Center for Systems Biology and Bioenergetics, Nijmegen Center for Mitochondrial Disorders, Radboud University Medical Center, Nijmegen, 6500 HB, The Netherlands

[3] Computational Discovery and Design Group, Center for Molecular and Biomolecular Informatics (CMBI), Radboud University Medical Center, Nijmegen, 6500HB, The Netherlands

[4] Department of Pediatrics, Radboud University Medical Center, Nijmegen, 6500HB, The Netherlands

[5] Department of Biochemistry, Radboud University Medical Center, Nijmegen, 6500HB, The Netherlands

***Correspondence to:**

Prof. Dr. Frans G.M. Russel,

Radboud University Medical Center

Department of Pharmacology and Toxicology

Geert Grooteplein 21

PO BOX 9101

6500 HB Nijmegen

The Netherlands

E-mail: frans.russel@radboudumc.nl

Supplementary Data

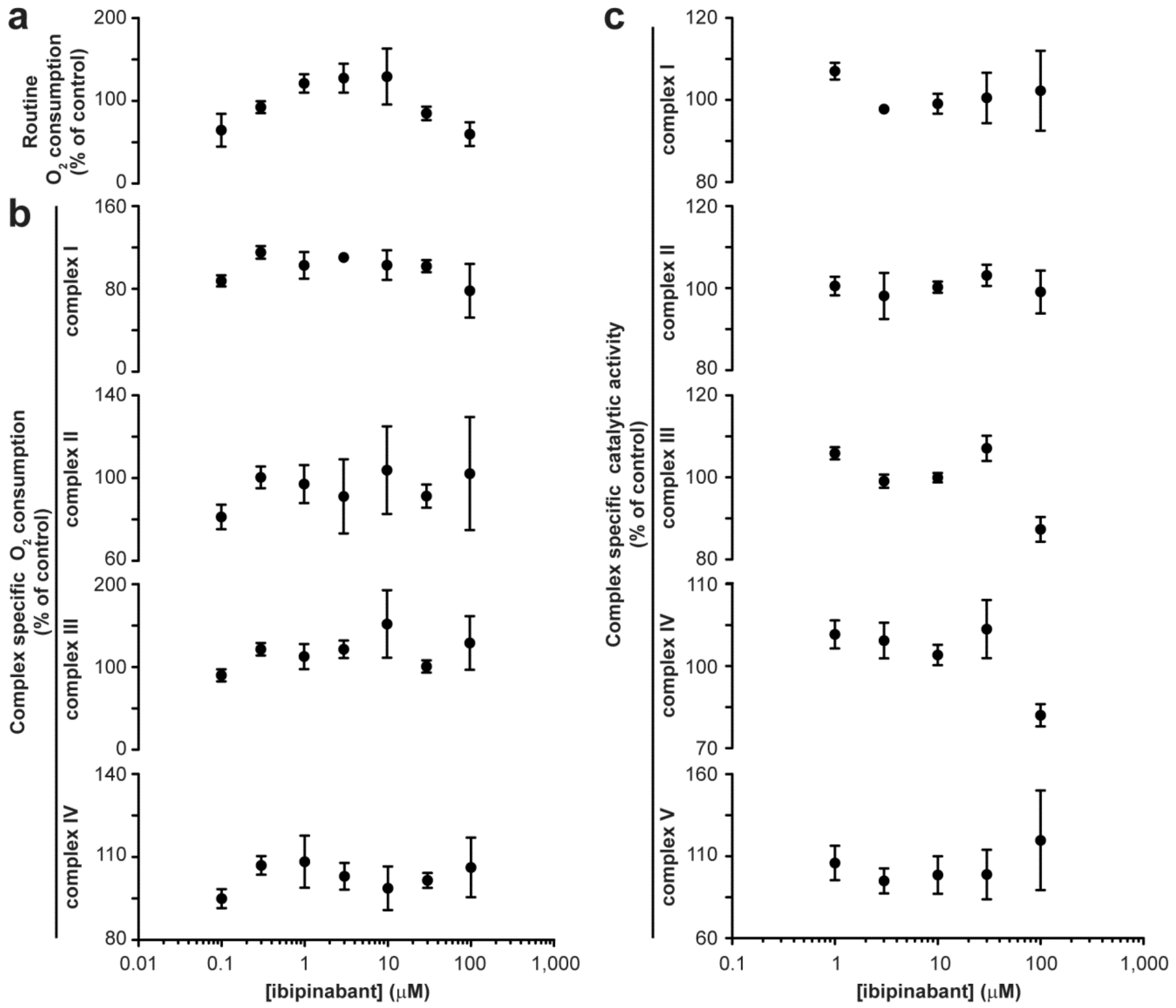

Supplementary Figure 1 | Ibipinabant-induced mitochondrial dysfunction is not linked to decreased respiratory capacity or direct inhibition of respiratory chain complexes. (A) Basal respiratory rates were determined after 4 hours of incubation with increasing ibipinabant concentrations. Data are expressed as percentage of vehicle (33±2 pmolO_2/s/10^6cells). Next to recording of basal respiratory rates, digitonin was added to the chambers of the respirometer to permeabilise the cells for measurement of **(B)** complex (C)I-, CII-, CIII-, and CIV-driven oxygen consumption. The data presented are expressed as percentage of vehicle-treated control (pmolO_2/s/10^6cells): 81±4 for CI, 92±4 for CII, 47±3 for CIII, 262±12 for CIV. **(C)** After 4 hours exposure of C2C12 myoblasts to increasing ibipinabant concentrations a mitochondrial-enriched fraction was prepared and freeze-thawed for permeabilisation of the mitochondrial membranes. The fractions were resuspended in the assay medium and the catalytic capacity of CI, CII, CIII, CIV, and CV was measured. The data presented are expressed as percentage of vehicle control: 190±30 mU/U CS for CI, 204±10 mU/U CS for CII, 560±30 mU/U CS for CIII, 360±40 mU/U CS for CIV, and 350±30 mU/U COX for CV. Statistical analysis: one-way ANOVA with Dunnett's post hoc analysis was applied to compare values to vehicle control, no statistically significant differences could be observed. Mean ± SEM; n=3 independent experiments.
